# Resin infiltration and oral health–related quality of life in children and adolescents: a narrative review

**DOI:** 10.3389/fdmed.2026.1795693

**Published:** 2026-05-21

**Authors:** Aruzhan Askarova, Raisa Uraz, Arnagul Kaliyeva, Azamat Omargali, Al Farabi Zhangabay, Gulzhan Tulegenova, Adilet Askarov, Nurgul Ablakimova

**Affiliations:** 1 Department of Dental Disciplines with Maxillofacial Surgery, West Kazakhstan Marat Ospanov Medical University, Aktobe, Kazakhstan; 2 Department of Children Diseases №2, West Kazakhstan Marat Ospanov Medical University, Aktobe, Kazakhstan; 3 Department of Surgical Diseases with Urology №2, West Kazakhstan Marat Ospanov Medical University, Aktobe, Kazakhstan; 4 Department of Pharmacology, Clinical Pharmacology, West Kazakhstan Marat Ospanov Medical University, Aktobe, Kazakhstan

**Keywords:** dental caries, dental enamel, minimally invasive surgical procedures, oral health-related quality of life, pediatric dentistry, resin infiltration, synthetic resins

## Abstract

**Background:**

Oral health plays a critical role in children's physical, emotional, and social development, yet traditional clinical indices do not adequately capture the broader impact of oral conditions on daily functioning and well-being. Oral health–related quality of life (OHRQoL) has therefore emerged as a key patient-centered outcome in pediatric dentistry.

**Methods:**

This narrative review aimed to summarize the conceptual foundations and measurement tools of OHRQoL in children and adolescents and to explore its relevance in minimally invasive caries management, with a particular focus on resin infiltration (RI) using Icon® (DMG, Hamburg, Germany). A targeted literature search was performed in PubMed/MEDLINE, Scopus, and Web of Science to identify relevant publications from 2000 to 2025. This narrative review was prepared in accordance with the Scale for the Assessment of Narrative Review Articles (SANRA), and the selected literature was discussed using a thematic approach.

**Results:**

The findings indicate that RI reduces procedural pain and anxiety, preserves tooth structure, and improves dental esthetics, particularly in visible teeth, outcomes that closely correspond to core OHRQoL domains such as functional limitations, emotional well-being, and social well-being. Although direct longitudinal studies assessing changes in validated pediatric OHRQoL scores following RI remain limited, substantial indirect evidence supports a plausible positive impact on children's quality of life.

**Conclusion:**

Current evidence indicates that RI has potential relevance to OHRQoL in children and adolescents, but direct clinical evidence remains insufficient. Future studies should incorporate validated OHRQoL measures to better determine the patient-centered benefits of RI and to support evidence-based pediatric dental care.

## 1 Introduction

Oral health is a crucial component of children's overall well-being, influencing their physical, psychological, social, and educational development (1). Dental caries (DC), malocclusion, traumatic dental injuries, and other oral conditions can cause pain, impair chewing, speech, and sleep, reduce self-confidence, and limit social participation (2). These consequences extend beyond clinical symptoms, affecting not only the child but also the family's quality of life. Traditional indices, such as the Decayed, Missing, and Filled Teeth (DMFT) index, provide valuable epidemiological data but fail to capture the subjective burden of oral diseases on daily functioning (3).

Oral diseases represent one of the most prevalent noncommunicable conditions worldwide, impacting approximately 45% of the global population, or nearly 3.5 billion individuals (4). In the United States, about 11% of children aged 2–5 years have at least one primary tooth with untreated decay, and that figure rises to nearly 18% among children aged 6–8 years. Nationwide data indicate that approximately 45.8% of those aged 2–19 years have experienced DC (treated or untreated), with 13.0% having untreated caries (5). An Indian evaluative study in preschoolers also found that increasing levels of caries were strongly correlated with poorer oral health-related quality of life among children and their families, as assessed using the Odia version of the Early Childhood Oral Health Impact Scale (ECOHIS) (6). These findings emphasize that oral diseases impose not only clinical but also significant psychosocial and economic consequences.

In recent decades, the concept of Oral Health–Related Quality of Life (OHRQoL) has emerged as a multidimensional construct designed to capture these broader impacts. OHRQoL reflects not only the presence or absence of disease but also its influence on physical functioning, emotional well-being, and social interaction. By addressing dimensions that traditional clinical measures overlook, OHRQoL offers a more comprehensive understanding of children's oral health status (7).

Parallel to the growing emphasis on patient-centered outcomes, pediatric dentistry has undergone a paradigm shift toward minimally invasive management of DC (8). Contemporary approaches prioritize early detection, preservation of sound tooth structure, and prevention of disease progression, while minimizing pain, anxiety, and treatment-related trauma (9). Such strategies are particularly important in children, for whom negative dental experiences may have long-lasting psychological and behavioral consequences, including dental fear and avoidance of care (10, 11).

Among minimally invasive interventions, resin infiltration (RI) using Icon® (DMG, Hamburg, Germany) has gained increasing attention as an innovative approach for the management of non-cavitated enamel lesions and white spot lesions (12). RI involves the application of a low-viscosity, light-cured resin infiltrant, primarily composed of triethylene glycol dimethacrylate (TEGDMA). Owing to its high penetration coefficient, the material infiltrates the porous structure of demineralized enamel via capillary action. By occluding microporosities and modifying the refractive index of the lesion, it contributes to both the arrest of early carious lesions and the improvement of esthetic appearance. By infiltrating the porous structure of demineralized enamel, RI aims to arrest lesion progression while simultaneously improving esthetics, without the need for drilling or local anesthesia (13). These characteristics suggest that RI may offer benefits that extend beyond clinical outcomes, potentially influencing children's comfort, self-esteem, treatment acceptance, and overall quality of life.

Despite the widespread clinical adoption of minimally invasive techniques such as RI, their broader impact on children's lived experiences remains insufficiently explored. In particular, it is unclear to what extent such interventions translate into measurable improvements in OHRQoL, and whether these benefits are sustained over time across different age groups and sociocultural settings.

The purpose of this article is therefore to summarize the conceptual foundations of OHRQoL in pediatric dentistry, to examine its relevance in the context of minimally invasive caries management, and to highlight the potential role of RI in improving patient-centered outcomes. By integrating clinical and psychosocial perspectives, this review aims to identify current evidence gaps and outline future directions for research and practice.

## 2 Materials and methods

This article was conducted as a narrative review focusing on the relationship between RI and OHRQoL in children and adolescents. A narrative review design was selected to allow a comprehensive and critical synthesis of heterogeneous evidence, including clinical studies, observational research, reviews, and conceptual papers, which could not be adequately addressed using formal systematic review or meta-analytical approaches. The conduct and reporting of the review were guided by the Scale for the Assessment of Narrative Review Articles (SANRA) to enhance methodological transparency, coherence, and scientific rigor (14). A.A., R.U., A.O., and A.F.Z. have clinical and academic experience relevant to pediatric dentistry, minimally invasive caries management, and child oral health. A.K. and G.T. contribute expertise in pediatric patient care and child health perspectives. A.A. and N.A. contributed methodological, analytical, and evidence-synthesis expertise relevant to the design, critical appraisal, and interpretation of this narrative review.

A structured literature search was performed in PubMed/MEDLINE, Scopus, and Web of Science, covering publications from January 2000 to December 2025. The search strategy was designed to capture literature related to RI and patient-centered outcomes in pediatric dentistry and was adapted for each database using combinations of controlled vocabulary and free-text terms. Key search concepts were combined using Boolean operators (AND, OR), for example: (“resin infiltration” OR “ICON”) AND (“oral health-related quality of life” OR “OHRQoL” OR “quality of life” OR “patient-reported outcomes” OR “patient satisfaction” OR “dental anxiety” OR “pain” OR “esthetic outcomes”) AND (“children” OR “adolescents” OR “pediatric dentistry”). Both controlled vocabulary (e.g., MeSH terms such as “Dental Caries”, “Quality of Life”, “Pediatric Dentistry” and “Synthetic Resins”) and free-text terms were applied to ensure broad coverage of relevant literature, including studies reporting outcomes conceptually related to OHRQoL. In addition, reference lists of relevant articles were manually screened to identify further publications of potential relevance.

Studies were considered eligible if they involved children or adolescents aged 18 years or younger and addressed RI as a clinical intervention. Particular emphasis was placed on publications reporting patient-centered outcomes, including OHRQoL, psychosocial impact, esthetic perception, treatment comfort, dental anxiety, or patient and parent satisfaction. Clinical trials, observational studies, narrative or systematic reviews, and conceptual or methodological papers published in English were eligible for inclusion. Studies conducted exclusively in adult populations, *in vitro* or animal studies without clinical or patient-reported outcomes, isolated case reports with limited outcome description, and conference abstracts without full-text availability were excluded. No restrictions were applied regarding comparator interventions or specific study designs, consistent with the narrative review methodology. Titles and abstracts identified through the search were screened for relevance, and full texts of potentially eligible publications were reviewed to confirm inclusion. In line with the narrative design, study selection was guided by conceptual relevance to the research question rather than by a predefined systematic selection framework. Data were extracted narratively, focusing on study characteristics, population and clinical indications for RI, type of outcome assessment, and key findings relevant to OHRQoL or related domains. Given the heterogeneity of study designs and outcome measures and the limited number of studies directly assessing OHRQoL following RI, data were synthesized descriptively rather than quantitatively.

The evidence was synthesized using a thematic narrative approach, integrating conceptual and empirical findings to explore the relationship between minimally invasive caries management and quality-of-life outcomes. This approach allowed integration of both empirical evidence and conceptually relevant findings related to patient-centered outcomes. Particular attention was given to studies employing validated OHRQoL instruments as well as to those reporting surrogate outcomes likely to influence quality of life, such as pain reduction, esthetic improvement, and treatment acceptability. In line with the narrative review design, formal risk-of-bias assessment and PRISMA reporting were not applied; instead, methodological strengths and limitations of the included studies were critically discussed to support balanced interpretation of the findings.

Given the narrative design and the inclusion of heterogeneous evidence, a formal tabular summary of individual studies was not constructed; instead, key findings are presented within the thematic sections of the manuscript.

## 3 Conceptual foundations of OHRQoL

The concept of OHRQoL reflects the understanding that oral health extends beyond the absence of disease and includes its influence on an individual's daily functioning and psychosocial well-being. The World Health Organization defines health as a state of complete physical, mental, and social well-being, and this principle underpins the development of OHRQoL as a multidimensional construct (15).

**Figure 1:**
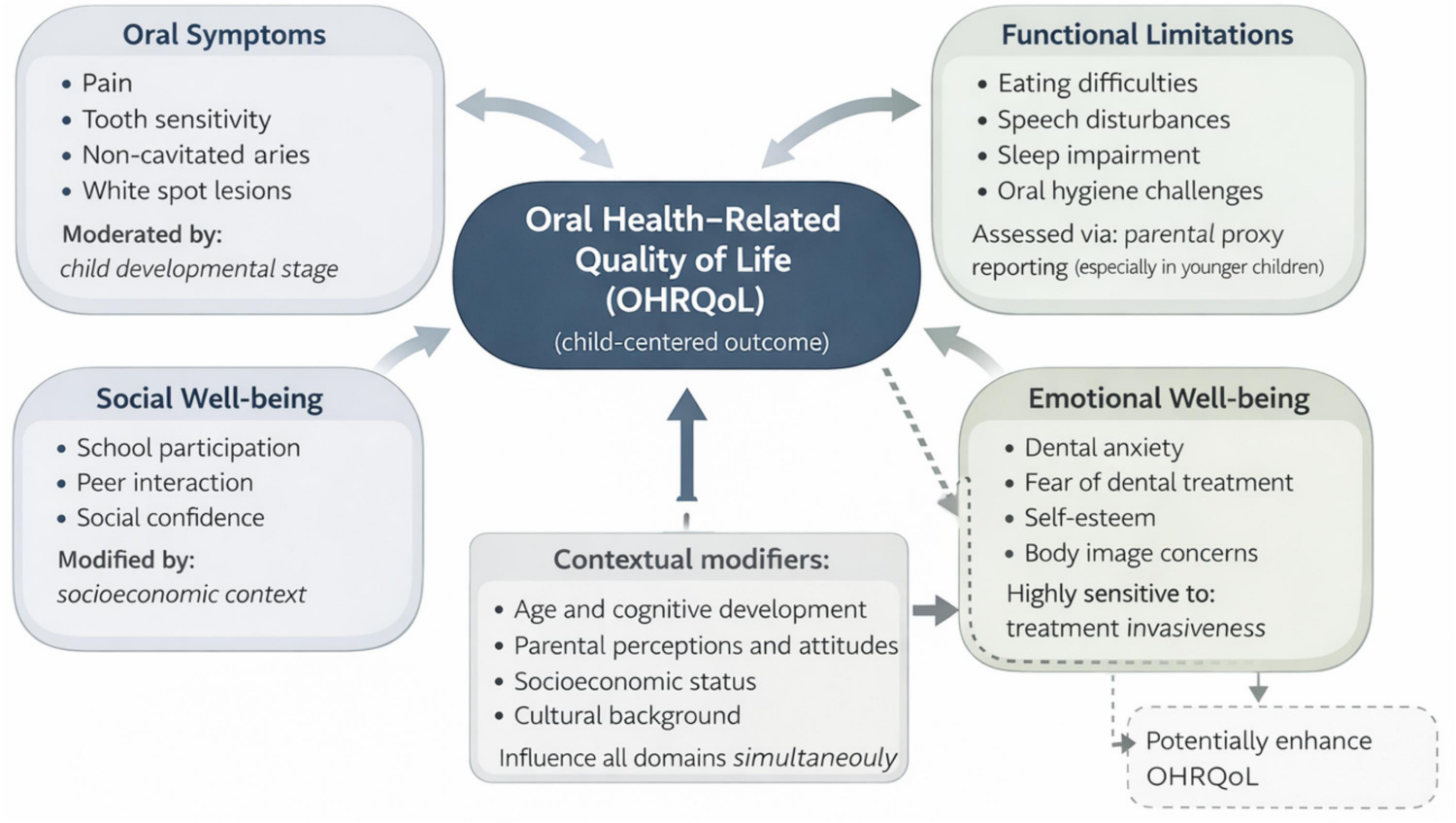
Conceptual framework of oral health–related quality of life in children. Original figure developed by the authors with AI-assisted drafting support (ChatGPT, OpenAI).

In children, OHRQoL encompasses four major domains: oral symptoms, functional limitations, emotional well-being, social well-being (Figure 1). Oral symptoms encompass experiences such as pain, tooth sensitivity, and halitosis, which interfere with eating and cause daily discomfort. Functional limitations include difficulties in chewing, speaking, sleeping, or maintaining oral hygiene, each of which can compromise normal development and general well-being. Emotional well-being reflects the psychological consequences of poor oral health, including fear of dental treatment, embarrassment about dental appearance, diminished self-esteem, and dissatisfaction with one's smile. Finally, social well-being captures the broader social impact, as children with oral health problems may struggle with peer interactions, experience reduced school performance or absenteeism, and face restrictions in participating in family and community activities. Taken together, these domains highlight that oral health extends beyond the clinical condition of the teeth and gums, influencing multiple aspects of children's daily lives (16).

Unlike adults, children's perception of oral health is influenced by their developmental stage, cognitive abilities, parental attitudes, and family socioeconomic background (17). This means that both self-reported and proxy-reported assessments are necessary to obtain a comprehensive picture. For preschool children, proxy measures such as parental questionnaires are often the most reliable, whereas school-aged children and adolescents are capable of self-reporting their experiences.

Thus, OHRQoL provides a framework that integrates biological, psychological, and social dimensions of oral health, offering a patient-centered perspective that complements traditional clinical indicators.

## 4 Measurement tools in pediatric dentistry

A variety of validated questionnaires have been developed to assess OHRQoL in children (18). These tools differ in terms of age-appropriateness, respondent type (child or parent), and scope of domains assessed. These instruments provide the methodological foundation for interpreting patient-centered outcomes in studies of minimally invasive interventions.

### 4.1 Child perceptions questionnaires (CPQ)

The Child Perceptions Questionnaires (CPQ) are among the most widely used instruments for school-aged children. Versions have been developed for specific age groups: CPQ8–10 for younger children and CPQ11–14 for older children and adolescents. Each questionnaire addresses four domains—oral symptoms, functional limitations, emotional well-being, and social well-being—allowing for a comprehensive evaluation of the child's oral health experience. CPQs are self-administered, but younger children may require assistance from trained interviewers to ensure comprehension and accuracy (19).

### 4.2 Parental perceptions questionnaire (PPQ)

For children unable to self-report reliably, parent-proxy measures are indispensable. The Parental Perceptions Questionnaire (PPQ) captures caregivers' perspectives on how oral conditions influence a child's quality of life. While proxy reports may not fully reflect children's subjective experiences, they are particularly valuable for preschool-aged children and those with developmental or cognitive limitations (20).

### 4.3 Early childhood oral health impact scale (ECOHIS)

ECOHIS was specifically designed for children aged 2–5 years. It assesses not only child-focused domains (such as pain, difficulty eating, or trouble sleeping) but also family-related consequences, including parental distress and financial impact. Its ability to capture both individual and family dimensions makes ECOHIS a widely adopted tool in pediatric oral health research (21).

### 4.4 Oral health impact profile (OHIP)

Although originally developed for adults, the Oral Health Impact Profile (OHIP) and its shorter versions (OHIP-14, OHIP-5) have occasionally been adapted for use in adolescents. These adaptations, while less common, provide a means to compare OHRQoL across different age groups (22).

Together, these instruments offer a versatile toolkit for evaluating OHRQoL, enabling clinicians and researchers to select measures best suited to the child's developmental stage and the study context. In general, their strengths lie in age-appropriate design, multidimensional coverage of symptoms and psychosocial outcomes, and the availability of both self- and proxy-report formats. At the same time, limitations include reliance on parental reporting in younger children, the need for adequate literacy and cognitive skills in older age groups, challenges of cultural adaptation, and variation in length and feasibility across tools. A comparative overview of the core and additional instruments is presented in Table 1.

**Table 1:** Comparison of OHRQoL measurement tools in pediatric dentistry.

Instrument	Target age group	Respondent	Domains assessed	Advantages	Limitations
Core Instruments					
Child Perceptions Questionnaire (CPQ8–10, CPQ11–14)	8–10 years, 11–14 years	Child (self-report)	Oral symptoms, functional limitations, emotional well-being, social well-being	Age-specific versions; widely validated; sensitive to change after treatment	Requires literacy and cognitive maturity; younger children may need assistance
Parental Perceptions Questionnaire (PPQ)	Mainly preschoolers	Parent (proxy-report)	Daily life impact from caregiver's perspective	Useful when children cannot self-report; captures caregiver insights	Parental bias; may not reflect child's subjective feelings
Early Childhood Oral Health Impact Scale (ECOHIS)	2–5 years	Parent (proxy-report)	Child symptoms and functions; family impacts (distress, financial burden)	Designed for preschoolers; includes family dimension; widely translated	Proxy-only; less sensitive to small longitudinal changes
Additional Instruments					
Family Impact Scale (FIS)	Children of various ages	Parent (proxy-report)	Emotional, financial, and social impacts on the family	Focuses on caregiver/family burden; complements child-centered tools	Does not capture child's perspective directly
Scale of Oral Health Outcomes for 5-year-olds (SOHO-5)	5-year-olds	Child (self-report) + parent-proxy version	Eating, sleeping, smiling, talking, emotional well-being	Simple pictorial design; suitable for very young children	Limited to age 5; narrow scope
Pediatric Oral Health-Related Quality of Life (POQL)	2–14 years	Child (self-report) and parent-proxy	Role function, physical function, social function, emotional function	Covers broad pediatric age range; dual versions	Less internationally validated than CPQ/ECOHIS
Child Oral Health Impact Profile (COHIP, COHIP-SF)	8–15 years	Child (self-report)	Oral health, functional well-being, emotional well-being, school environment, self-image	Comprehensive; short-form available; useful in orthodontics/craniofacial research	Long version time-consuming; not for very young children
Michigan Oral Health-Related QoL (MOHRQoL)	Preschoolers	Parent (proxy-report)	Early child oral health impacts	Early pediatric-specific measure	Rarely used now; largely replaced by ECOHIS
Scale of Oral Health Outcomes for 5-year-olds (SOHO-5)	5-year-olds	Child (self-report) + parent-proxy version	Eating, sleeping, smiling, talking, emotional well-being	Simple pictorial design; suitable for very young children	Limited to age 5; narrow scope

## 5 Cross-cultural adaptation of OHRQoL instruments

The global application of OHRQoL instruments requires rigorous cross-cultural adaptation. Tools such as the ECOHIS and the CPQ were originally developed in English, but their relevance across diverse populations depends on careful translation, cultural sensitivity, and psychometric validation. Cross-cultural adaptation extends beyond linguistic equivalence, ensuring that items adequately capture culturally specific perceptions of oral health and quality of life.

In India, ECOHIS has been translated and validated in several regional languages, including Hindi, Tamil, and Odia. The Hindi version showed excellent internal consistency (Cronbach's *α* = 0.87) and test–retest reliability (ICC = 0.91) (23), while the Odia version demonstrated acceptable reliability (Cronbach's *α* ≈ 0.71) and construct validity, although some items required cultural reinterpretation to maintain conceptual accuracy (6).

In Brazil, the Portuguese version (B-ECOHIS) has undergone extensive validation (24) and has been widely applied in both national surveys and clinical studies. Importantly, it has shown high responsiveness to improvements in OHRQoL following DC treatment and preventive interventions (25), making it a reference model for adaptation in other cultural contexts.

Across Europe, the German version of ECOHIS showed strong internal consistency (Cronbach's *α* > 0.80) and test–retest reliability (26). In Turkey, a Turkish ECOHIS adaptation proved reliable and responsive to clinical changes after dental treatment (27).

In Asia, the Chinese ECOHIS demonstrated high internal consistency and good convergent validity, and has been widely applied in epidemiological surveys (28); The Malay ECOHIS in Malaysia confirmed satisfactory reliability and validity in preschool children (29).

Latin American countries beyond Brazil have also contributed to adaptation: the Chilean version of ECOHIS confirmed reliability and validity among preschool children (30), and the Mexican Spanish version of CPQ11-14 has been validated for school populations (31).

By contrast, in Central Asia—including Kazakhstan—locally validated versions of ECOHIS remain scarce. Russian-language adaptations are sometimes employed, but they were primarily developed in other countries and may not fully reflect local sociocultural realities. Given Kazakhstan's bilingual environment, with both Kazakh and Russian widely spoken, there is a pressing need for dual-language validation studies that adequately capture cultural perceptions of financial burden, emotional distress, and school-related impacts of oral conditions. Addressing this gap would not only improve the accuracy of OHRQoL assessment in Central Asia but also enhance the comparability of findings across international studies (30, 32).

## 6 Socioeconomic inequalities and OHRQoL

Beyond linguistic and cultural factors, children's OHRQoL is also profoundly shaped by broader social determinants of health. Socioeconomic status influences not only access to dental care, but also the lived experience of oral disease captured by OHRQoL instruments.

Socioeconomic status is one of the strongest determinants of both oral health and OHRQoL in children. Lower parental education, limited household income, and poor access to preventive dental services consistently correlate with worse OHRQoL outcomes (33). Children from disadvantaged backgrounds are more likely to experience untreated DC, malocclusion, and traumatic dental injuries, which in turn impair daily functioning, reduce school performance, and negatively affect social and emotional well-being (34).

A large household survey in rural Egypt found that caries experience and self-perceived oral health substantially reduced OHRQoL for both children and their mothers, illustrating the intergenerational impact of poverty on oral health (2). Similarly, studies from India and Brazil report that children from low-income families have worse OHRQoL scores, reflecting greater functional limitations and family-related distress (17, 24). These observations are in line with the World Health Organization's Global Oral Health Status Report (2022), which highlights the disproportionate burden of oral diseases among socioeconomically deprived populations and the need to address social determinants in oral health policy (4).

Overall, the evidence indicates that OHRQoL instruments are sensitive not only to clinical disease but also to broader social determinants of health; thus, they are powerful tools for documenting inequalities and advocating for targeted public health interventions (17, 33). Incorporating OHRQoL measures into epidemiological surveys and national oral health programmes can reveal hidden disparities and guide policies to ensure that improvements in oral health are equitably distributed across populations (35).

## 7 Minimally invasive approaches in pediatric dentistry

Minimally invasive dentistry (MID) has become a cornerstone of contemporary pediatric caries management, reflecting a shift from surgically oriented treatment toward biologically driven, patient-centered care. This paradigm emerged from advances in cariology demonstrating that DC is a dynamic, biofilm-mediated process that can be controlled without extensive removal of tooth structure. Frencken et al. defined MID as an approach that prioritizes early lesion detection, risk-based prevention, and preservation of sound dental tissues (36), while Schwendicke et al. further emphasized that the degree of invasiveness should be minimized whenever possible to reduce biological and psychological harm (37, 38).

In pediatric populations, the rationale for MID extends beyond tissue preservation. Children are particularly susceptible to pain, fear, and stress associated with dental procedures, and these experiences are strongly linked to the development of dental anxiety and long-term avoidance of dental care. Recent longitudinal evidence indicates that dental fear trajectories established in childhood persist into adolescence, and that early negative dental experiences are associated with higher levels of dental fear later in life (39, 40). Consequently, reducing procedural invasiveness is not merely a technical consideration but a critical component of preventive oral health strategies in children.

A wide range of minimally invasive approaches is currently used in pediatric dentistry, including non-operative preventive strategies (fluoride varnishes, fissure sealants), remineralization therapies (fluoride- or calcium–phosphate-based agents), chemotherapeutic approaches such as silver diamine fluoride (SDF), and minimally invasive restorative techniques such as atraumatic restorative treatment (ART). Clinical studies comparing ART with conventional restorative procedures consistently report lower pain perception, improved child cooperation, and reduced need for behavioral management in the ART group (41, 42). These findings suggest that minimizing procedural burden can substantially improve the child's treatment experience.

From a patient-centered perspective, MID has also been linked to improvements in OHRQoL. Novaes et al. demonstrated that the responsiveness of ECOHIS was strongly related to treatment complexity, with less invasive procedures producing greater improvements in both child- and family-related domains (43). Similarly, a systematic review and meta-analysis by Aimée et al. confirmed that OHRQoL instruments are sensitive to caries-related interventions, particularly those that reduce pain, functional limitations, and psychosocial distress (35). Preventive and community-based programs further support this association; Alsumait et al. showed that school-based preventive interventions were associated with improved parent-reported OHRQoL alongside better clinical outcomes (44).

However, MID approaches are not uniform in their psychosocial impact. While SDF is highly effective in arresting caries, studies by Crystal and Niederman, as well as Ruff and Niederman, highlight that esthetic concerns—especially discoloration of anterior teeth—may negatively influence parental satisfaction and psychosocial domains of OHRQoL (45, 46). These findings underscore the importance of evaluating minimally invasive methods not only in terms of biological efficacy but also with respect to their broader impact on children's daily lives.

## 8 Resin infiltration: principles and components

RI was developed as a microinvasive technique to address the limitations of both non-operative preventive strategies and conventional restorative treatment. The scientific basis of RI lies in the recognition that early enamel caries is characterized by subsurface demineralization beneath a relatively intact surface layer, which impedes the penetration of remineralizing agents and allows lesion progression. Paris and Meyer-Lueckel demonstrated that low-viscosity resin infiltrants can penetrate these porous lesions and effectively inhibit lesion progression by occluding diffusion pathways (38, 47).

In a systematic review of non-cavitated caries lesions, concluded that RI was more effective than non-invasive measures alone in arresting proximal enamel caries (48). Subsequent randomized clinical trials and long-term prospective studies have confirmed sustained lesion stabilization following RI, supporting the biological plausibility and clinical durability of this microinvasive approach (49–51).

**Figure 2:**
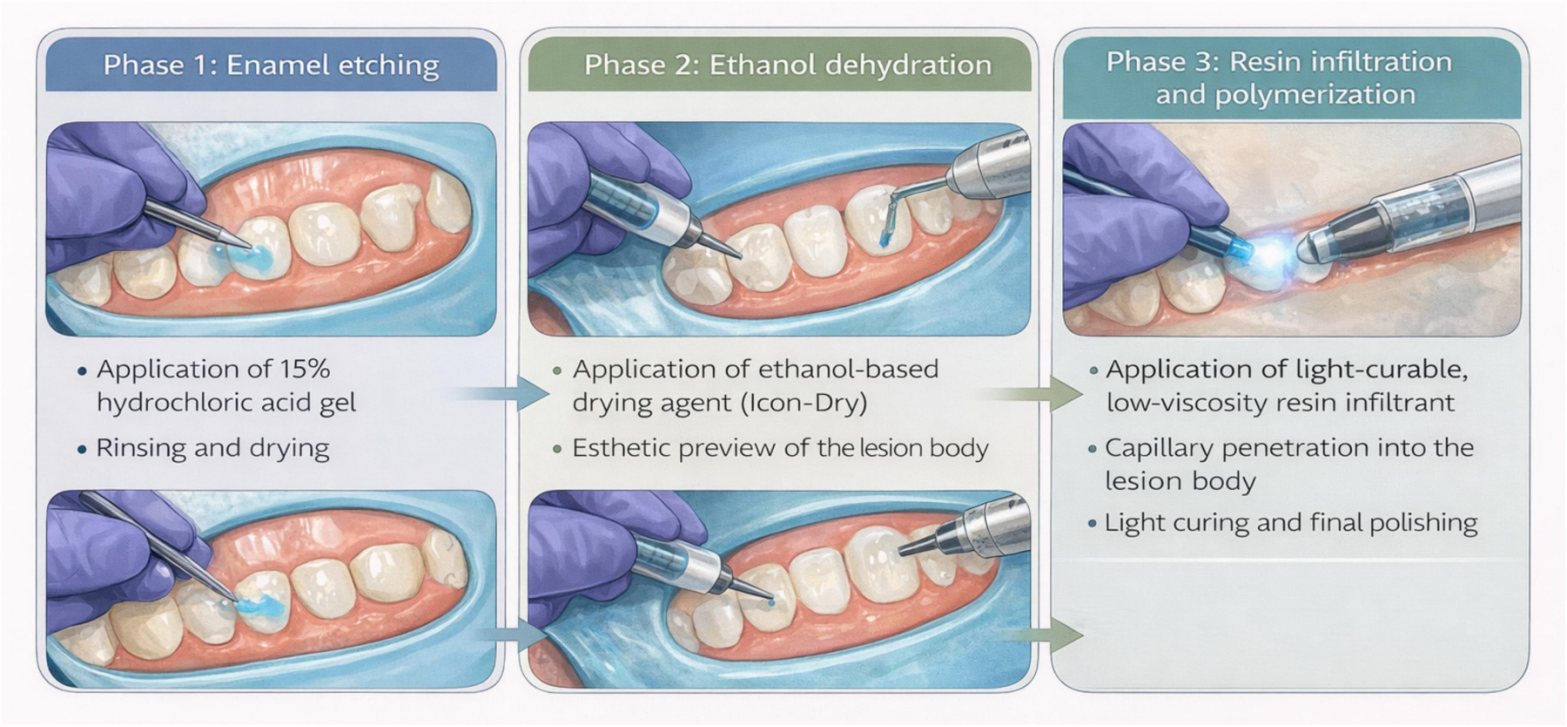
Resin infiltration using icon® (DMG, Hamburg, Germany): three key procedural phases for smooth-surface enamel lesions. Original schematic illustration created by the authors based on the manufacturer's instructions, with AI-assisted drafting support (ChatGPT, OpenAI).

The clinical procedure for RI, based on the manufacturer's instructions, comprises three key steps. First, the tooth is isolated, typically using a rubber dam, to ensure a dry working field, and a hydrochloric acid-based etchant is applied to remove the superficial pseudo-intact enamel layer, thereby exposing the lesion body. Second, ethanol dehydration enhances resin penetration and allows visual assessment of the anticipated esthetic outcome. Finally, a light-curable, low-viscosity resin is applied, penetrating the lesion body via capillary action and subsequently polymerized to form a stable barrier. Excess material is removed prior to light curing, and in some cases, a second application may be performed to improve infiltration and esthetic outcomes. This process not only arrests lesion progression but also modifies the optical properties of enamel by approximating the refractive index of healthy tissue (Figure 2).

Clinically, RI occupies an intermediate position within the MID spectrum. It is more invasive than topical remineralization but substantially less invasive than conventional restorative treatment, as it avoids mechanical preparation and removal of sound tooth structure. This unique position has contributed to its growing adoption, particularly for early proximal lesions and post-orthodontic white spot lesions.

## 9 Relevance of resin infiltration for children and adolescents

The relevance of RI for pediatric and adolescent patients is underpinned by biological, psychological, and social considerations. Biologically, children often present with early-stage lesions that are ideally suited for infiltration, offering an opportunity to intervene before cavitation and the restorative cycle begin (52). Early stabilization of lesions may reduce the cumulative restorative burden over the child's lifetime.

Psychologically, RI addresses key determinants of treatment-related fear and anxiety. Avoidance of drilling and local anesthesia significantly reduces procedural discomfort and perceived threat, which are major contributors to dental anxiety in children (53, 54). Paris and Meyer-Lueckel highlighted that the minimally invasive nature of RI makes it particularly suitable for young patients and those with limited tolerance for conventional procedures (55).

Adolescents represent a subgroup for whom the esthetic benefits of RI are especially salient. Post-orthodontic white spot lesions have been associated with dissatisfaction, reduced self-confidence, and social embarrassment. Several studies have documented the esthetic effectiveness of RI in the management of post-orthodontic white spot lesions. Kim et al., Knosel et al. reported significant and sustained improvements in enamel appearance following RI, with high levels of patient satisfaction observed during follow-up periods extending beyond one year (56, 57). Similarly, dos Santos and colleagues demonstrated that RI yielded superior esthetic outcomes when compared with other microinvasive approaches, including remineralizing agents, particularly for lesions located in the esthetic zone (58). Although these investigations primarily focused on esthetic endpoints and subjective satisfaction rather than validated OHRQoL instruments, the affected outcomes closely correspond to the emotional and social domains commonly incorporated into quality-of-life frameworks.

Beyond immediate outcomes, positive experiences with minimally invasive treatments may influence long-term oral health behaviors. Children who undergo painless, well-tolerated procedures are more likely to develop trust in dental professionals and engage with preventive care, supporting sustained oral health benefits.

Importantly, the clinical indications for RI are heterogeneous and may differ in their potential impact on OHRQoL. For example, post-orthodontic white spot lesions and other visible enamel defects, particularly in the anterior region, are likely to have a greater influence on emotional and social domains due to esthetic concerns and their effect on self-esteem and social interactions (59, 60). In contrast, non-cavitated proximal or early enamel caries lesions, which are often not visible, may have a more limited direct psychosocial impact but may still contribute to improved OHRQoL indirectly through enhanced treatment experience, reduced need for invasive procedures, and prevention of disease progression (61). These differences highlight the importance of considering the clinical context when interpreting the patient-centered impact of RI.

## 10 Resin infiltration and OHRQoL: conceptual and clinical links

OHRQoL provides a comprehensive framework for capturing the functional, emotional, and social consequences of oral disease and its management. Locker's conceptual model emphasizes that clinical indicators alone cannot adequately reflect the lived experience of oral health, a notion reinforced by subsequent empirical research (62).

Although direct evidence linking RI to changes in validated pediatric OHRQoL scores remains limited, a robust body of indirect evidence supports a plausible and clinically meaningful association. This indirect evidence framework is based on the alignment between well-documented clinical and psychosocial outcomes of resin RI and the core domains measured by pediatric OHRQoL instruments. Importantly, the magnitude and nature of these effects may vary depending on the specific clinical indication, particularly with regard to lesion visibility and its psychosocial relevance.

RI has consistently been shown to reduce procedural pain and discomfort, minimize treatment-related anxiety by avoiding drilling and local anesthesia, preserve tooth structure, and improve dental esthetics, particularly in visible anterior teeth. It should be noted that, in this context, pain reduction primarily refers to decreased procedural discomfort compared with conventional restorative treatment, rather than pain related to the lesion itself, which is typically absent in early-stage enamel lesions (63, 64). Each of these outcomes corresponds closely to established OHRQoL domains, including functional limitations, emotional well-being, and social well-being, as defined in widely used instruments such as ECOHIS and CPQ. These indirect links between RI-related outcomes and core OHRQoL domains are summarized in Table 2.

**Table 2:** Indirect links between clinical outcomes of resin infiltration and OHRQoL domains.

Clinical/psychosocial outcome of RI	Mechanism of action	Corresponding OHRQoL domain(s)	Expected impact on OHRQoL
Reduced procedural pain and discomfort	Avoidance of drilling and local anesthesia	Functional limitations	Improved eating, sleeping, and daily functioning
Reduced treatment-related anxiety and fear	Minimally invasive, non-threatening procedure	Emotional well-being	Lower dental fear, improved treatment acceptance
Preservation of sound tooth structure	Microinvasive lesion stabilization without cavity preparation	Functional limitations	Reduced long-term functional impairment
Arrest of lesion progression	Occlusion of diffusion pathways within enamel	Oral symptoms	Reduced sensitivity and discomfort
Improvement of dental esthetics (white spot lesions)	Optical masking and refractive index matching	Emotional well-being; Social well-being	Enhanced self-esteem, reduced embarrassment

RI, resin infiltration; OHRQoL, oral health–related quality of life.

Despite increasing clinical interest in resin infiltration, the number of original studies directly evaluating quality-of-life outcomes remains limited. To provide a structured overview of the available evidence, studies on RI and quality-of-life-related outcomes are summarized in Table 3.

**Table 3:** Selected clinical studies on resin infiltration with potential relevance to OHRQoL-related outcomes.

Study	Population	Study design	Main clinical outcome	Potential relevance to OHRQoL
Gallo et al., 2026 (70)	Children with MIH	Single-arm clinical study	Improved esthetics and reduced bullying	Direct relevance to psychosocial well-being
Knosel et al., 2019 (56)	Adolescents (post-orthodontic WSL)	Prospective study	Sustained esthetic improvement	Potential impact on self-esteem and social interactions
Arslan et al., 2020 (49)	Adolescents	RCT	Arrest of proximal caries lesions	Potential reduction in need for invasive treatment
Jorge et al., 2019 (50)	Children (primary molars)	RCT	Long-term lesion stabilization	Potential prevention of disease progression and related functional limitations
Kim et al., 2010 (57)	Children/adolescents with enamel defects	Observational clinical study	Improvement in anterior tooth esthetics	Potential impact on self-esteem and social perception

MIH, molar–incisor hypomineralization; WSL, white spot lesions; RCT, randomized controlled trial; RI, resin infiltration; OHRQoL, oral health–related quality of life.

Therefore, even in the absence of large longitudinal trials directly measuring OHRQoL changes after RI therapy, the convergence of clinical effectiveness, improved treatment experience, and esthetic benefits provides a strong conceptual and empirical rationale for expecting positive effects on children's OHRQoL.

At the same time, the underlying mechanism and limitations of RI should be considered when interpreting its potential impact on OHRQoL. RI is based on the penetration of a low-viscosity resin into the porous structure of demineralized enamel, resulting in occlusion of microporosities and modification of the refractive index of the lesion (55). While this mechanism contributes to the arrest of early carious lesions and improvement of esthetic appearance, several limitations should be acknowledged. The technique is primarily indicated for non-cavitated lesions and may be less effective in more advanced caries. Incomplete lesion masking has been reported, particularly in deeper or heterogeneous lesions. In addition, long-term color stability and potential discoloration remain concerns. The procedure is technique-sensitive and requires adequate isolation, and soft tissue irritation due to acid etching may occur (56, 60).

Systematic reviews and meta-analyses have demonstrated that pediatric OHRQoL instruments such as ECOHIS and CPQ are responsive to dental interventions, particularly those that reduce pain, functional impairment, and psychosocial burden (65–67).

Recent pediatric studies have demonstrated that improvements in comfort and dental esthetics following minimally invasive dental treatment are associated with significant gains in OHRQoL, even when clinical changes are modest. Randomized and prospective studies in preschool children have shown that non- and micro-invasive caries management strategies, such as SDF and fluoride-based interventions, lead to meaningful improvements in both child- and family-reported OHRQoL as assessed using validated instruments including ECOHIS (68, 69).

Although evidence directly evaluating changes in validated OHRQoL scores following RI in children and adolescents remains limited, emerging data and strong indirect evidence support a plausible association. Recent observational and pilot clinical studies in school-aged children and adolescents with enamel defects and early caries lesions report improvements in esthetic perception, social confidence, and reductions in psychosocial burden following resin infiltrant therapy, outcomes that closely correspond to core OHRQoL domains (45, 46).

RI targets multiple determinants of OHRQoL by minimizing pain and discomfort, reducing treatment-related anxiety, preserving tooth structure, and improving dental appearance. Compared with other minimally invasive approaches such as SDF—where esthetic drawbacks may negatively affect psychosocial domains – RI offers a combination of biological effectiveness and esthetic benefit that may be particularly advantageous for school-aged children and adolescents (45, 70).

The absence of robust, longitudinal pediatric OHRQoL data following RI represents a critical gap in the literature. Future studies integrating validated pediatric OHRQoL instruments, such as ECOHIS and CPQ, into clinical trials and observational research on RI would enable a more comprehensive evaluation of its patient-centered benefits and facilitate comparison with other minimally invasive strategies. Such research would support the broader transition toward value-based, child-centered pediatric dental care, in which success is defined not only by lesion control but also by improvements in children's daily functioning, psychosocial well-being, and social participation.

For clarity, it is important to distinguish between direct evidence (studies using validated OHRQoL instruments) and indirect or conceptually relevant evidence (studies reporting patient-centered outcomes aligned with OHRQoL domains). In this review, both types of evidence are considered, with explicit recognition of their different levels of inference. However, conclusions based on indirect associations should be interpreted with caution. This highlights the need for future studies directly evaluating the impact of RI on OHRQoL using validated pediatric instruments.

## 11 Economic and equity considerations of minimally invasive interventions in pediatric dentistry

While minimally invasive interventions are increasingly promoted as patient-centered and biologically conservative, their real-world impact is strongly influenced by economic and equity considerations. In pediatric dentistry, affordability, reimbursement structures, and access to trained providers play a critical role in determining whether minimally invasive methods translate into population-level benefits rather than remaining limited to selected clinical settings (70–72).

RI is often perceived as a relatively costly micro-invasive technique because of higher upfront material costs and chairside time compared with conventional preventive approaches. Economic evaluations conducted primarily in European settings indicate that the per-lesion material cost of RI may be approximately three to five times higher than that of fluoride varnish or SDF at the point of care (73). These higher initial costs may limit the routine implementation of RI in public dental services and low-resource settings, particularly in health systems where preventive and micro-invasive procedures are inadequately reimbursed.

However, focusing exclusively on short-term costs may substantially underestimate the long-term economic value of RI. Health economic modeling studies in cariology consistently demonstrate that early interception of non-cavitated caries lesions can reduce cumulative treatment costs over the life course by delaying or preventing entry into the restorative cycle (73, 74). Long-term models suggest that managing proximal enamel lesions using micro-invasive strategies is more cost-effective than operative treatment when evaluated over extended time horizons (73).

Evidence from modeling and clinical studies further suggests that micro-invasive approaches, including RI, can substantially reduce the need for operative treatment in selected early lesions, thereby decreasing downstream costs related to restorations, retreatment, and complications (37, 51). In pediatric populations, these savings may be amplified by a reduced need for pharmacological behavior management, sedation, or general anesthesia, which represent major direct and indirect healthcare costs (75).

From a quality-of-life perspective, economic considerations intersect closely with psychosocial outcomes. Although SDF is highly cost-effective and effective in arresting caries, its characteristic black discoloration has been associated with reduced parental acceptance and negative esthetic perceptions, particularly for anterior teeth, potentially affecting psychosocial domains of OHRQoL (45, 46). In contrast, RI provides esthetic improvement in addition to lesion control, which may confer added value in terms of emotional well-being and social participation, especially for school-aged children and adolescents (56).

Equity considerations are therefore central to the evaluation of minimally invasive interventions. While RI offer superior esthetic and patient-centered outcomes, higher upfront costs and limited reimbursement may restrict access predominantly to families of higher socioeconomic status or private care settings, raising concerns that advanced micro-invasive techniques could inadvertently widen oral health inequalities if not supported by appropriate policy measures (70).

Integrating economic evaluation alongside OHRQoL assessment in future pediatric studies of RI would allow a more comprehensive assessment of value, capturing not only clinical effectiveness but also affordability, acceptability, and long-term impact on children's quality of life. Such an approach aligns with emerging principles of value-based healthcare and is essential for the equitable implementation of minimally invasive pediatric dentistry across diverse socioeconomic and cultural contexts.

## 12 Future directions

Despite substantial advances in minimally invasive pediatric dentistry and in the conceptual understanding of OHRQoL, important gaps remain at the interface between clinical innovation, patient-reported outcomes, and implementation research. Addressing these gaps is essential to advance evidence-based, patient-centered care for children and adolescents (16).

One of the most critical future directions is the systematic integration of validated OHRQoL measures into clinical studies evaluating minimally invasive caries management strategies, including RI. Although numerous clinical trials and observational studies have demonstrated the biological effectiveness and esthetic benefits of RI, its impact on children's daily functioning, emotional well-being, and social participation has not been directly quantified using validated pediatric OHRQoL instruments (48). Given that tools such as ECOHIS and CPQ have repeatedly shown sensitivity to changes in pain, comfort, and psychosocial burden following dental interventions, their inclusion as secondary or co-primary outcomes in RI studies is both methodologically justified and clinically relevant (76).

Another important challenge concerns the heterogeneity of outcomes reported in pediatric dental research. While clinical endpoints such as lesion arrest, colorimetric changes, or surface integrity dominate studies of resin infiltration, patient-centered outcomes are inconsistently assessed. The development and adoption of standardized core outcome sets for pediatric dentistry would facilitate comparability across trials and strengthen the evidence base for synthesis (35).

Digital transformation offers additional opportunities to enhance both research and clinical practice. The transition from paper-based questionnaires to electronic patient-reported outcome measures (ePROMs) enables real-time data collection, improves feasibility of repeated assessments, and facilitates integration of OHRQoL evaluation into routine clinical workflows (77).

A further emerging direction lies in the integration of OHRQoL assessment with artificial intelligence–based diagnostic tools. Recent studies have demonstrated promising performance of AI-driven systems for early caries and white spot lesion detection using radiographic and intraoral imaging data (78).

Ensuring cultural and developmental appropriateness of OHRQoL assessment also remains a global priority, particularly in underrepresented regions, including Central Asia (30).

Ultimately, future research should align with the broader transition toward value-based pediatric dental care, in which success is defined not only by clinical effectiveness but also by improvements in children's daily functioning and overall well-being (79).

## 13 Conclusion

OHRQoL has become an essential component of pediatric dentistry, providing a patient-centered perspective that complements traditional clinical indices and captures the functional, emotional, and social consequences of oral disease. The growing body of evidence demonstrates that OHRQoL measures are sensitive to dental conditions and interventions in children and adolescents, supporting their integration into both clinical practice and research.

MID represents a paradigm shift toward biologically conservative and psychologically supportive care for pediatric patients. Within this framework, RI occupies a unique position by combining early caries control with preservation of tooth structure, avoidance of drilling and local anesthesia, and immediate esthetic improvement. These characteristics align closely with key determinants of OHRQoL, including comfort, emotional well-being, and social functioning.

Although direct evidence linking RI to changes in validated pediatric OHRQoL scores remains limited, available clinical and indirect data support a plausible positive impact on children's quality of life. This narrative review highlights a critical gap in the current literature and underscores the need for future studies integrating standardized OHRQoL assessment into evaluations of minimally invasive interventions.

## References

[1] Zaror C Matamala-Santander A Ferrer M Rivera-Mendoza F Espinoza-Espinoza G Martinez-Zapata MJ. Impact of early childhood caries on oral health-related quality of life: a systematic review and meta-analysis. Int J Dent Hyg. (2022) 20(1):120–35. 10.1111/idh.12494 33825317

[2] Aly NM Ihab M Ammar N Quritum M Moussa H El Tantawi M. Impact of dental caries and self-perceived oral health on daily lives of children and mothers in rural Egypt: a household survey. BMC Oral Health. (2024) 24(1):884. 10.1186/s12903-024-04454-9 39095790 PMC11297685

[3] Rodakowska E Jamiolkowski J Baginska J Kaminska I Gabiec K Stachurska Z Oral health-related quality of life and missing teeth in an adult population: a cross-sectional study from Poland. Int J Environ Res Public Health. (2022) 19(3):1626. 10.3390/ijerph19031626 35162649 PMC8834766

[4] World Health Organization. Global Oral Health Status Report: Towards Universal Health Coverage for Oral Health by 2030. Geneva: World Health Organization (2022).

[5] Fleming E Afful J. Prevalence of Total and Untreated Dental Caries among Youth: United States, 2015-2016. NCHS Data Brief. (2018) 307:1–8. 29717975

[6] Patyal N Rath H Mahapatra S. Impact of caries experience on the oral health-related quality of life of pre-school children and their families in an Indian city - an evaluative study. Indian J Dent Res. (2024) 35(2):136–9. 10.4103/ijdr.ijdr_928_21 39282761

[7] Das A Chellappa LR Indiran MA. Enhancing pediatric oral health-related quality of life: a comprehensive systematic review of oral health initiatives with practical applications and global relevance. J Pioneer Med Sci. (2025) 14(Suppl 1):89–97. 10.47310/jpms202514S0112

[8] Dhonde S Jawdekar A. Development of strategies based on the recent evidence for the management of dental caries in children: a systematic overview. In: Zabokova Bilbilova E, editor. Dental Caries – From Prevention to Restoration. London: IntechOpen (2025). 10.5772/intechopen.1011683

[9] Laureano ICC Farias L Fernandes LHF Cavalcanti AL. Prevalence of dental fear and its association with painful oral conditions in adolescents. Pesqui Bras Odontopediatria Clin Integr. (2023) 23:e230195. 10.1590/pboci.2023.075

[10] Cademartori MG Mathias FB Jansen K Goettems ML. Dental fear/anxiety in children and child emotional and behavioural problems. Pesqui Bras Odontopediatria Clin Integr. (2023) 23:e210226. 10.1590/pboci.2023.012

[11] Petkova-Ninova V Mitova N Bakardjiev P Georgieva M. Phobia, fear, and anxiety of dental treatment in pediatric patients: etiology and clinical implications. Probl Dent Med. (2025) 51:1–8. 10.3897/pdm.51.e167362

[12] Ekstrand K Martignon S Bakhshandeh A Ricketts DN. The non-operative resin treatment of proximal caries lesions. Dent Update. (2012) 39(9):614–6. 8-20, 22. 10.12968/denu.2012.39.9.614 23479850

[13] Abd Elkader D Ismail H Mowafy M Abousheleib M. Management of white spot lesions using resin infiltration and microabrasion: a comparative *in vitro* study. Egypt Orthod J. (2019) 56(December 2019):30–8. 10.21608/eos.2019.77633

[14] Baethge C Goldbeck-Wood S Mertens S. SANRA—a scale for the quality assessment of narrative review articles. Res Integr Peer Rev. (2019) 4(1):5. 10.1186/s41073-019-0064-8 30962953 PMC6434870

[15] Hescot P. The new definition of oral health and relationship between oral health and quality of life. Chin J Dent Res. (2017) 20(4):189–92. 10.3290/j.cjdr.a39217 29181455

[16] Thomson WM Broder HL. Oral-health-related quality of life in children and adolescents. Pediatr Clin North Am. (2018) 65(5):1073–84. 10.1016/j.pcl.2018.05.015 30213350

[17] Kumar S Kroon J Lalloo R. A systematic review of the impact of parental socio-economic status and home environment characteristics on children’s oral health related quality of life. Health Qual Life Outcomes. (2014) 12(1):41. 10.1186/1477-7525-12-41 24650192 PMC4000002

[18] Chai HH Gao SS Chen KJ Lo ECM Duangthip D Chu CH. Tools evaluating child oral health-related quality of life. Int Dent J. (2024) 74(1):15–24. 10.1016/j.identj.2023.07.004 37482502 PMC10829350

[19] Riou MC Bourmaud A Boizeau P de La Dure-Molla M Boy-Lefevre ML Friedlander L. Translation and validation of the French version of the child perceptions questionnaire for children aged 11 to 14 years old (Cpq11-14) short-form. Clin Oral Investig. (2024) 28(7):403. 10.1007/s00784-024-05793-1 38940970

[20] Procopio SW Tavares MC Carrada CF Ribeiro Scalioni FA Ribeiro RA Paiva SM. Perceptions of parents/caregivers about the impact of oral conditions on the quality of life of children and adolescents with autism spectrum disorder. J Autism Dev Disord. (2024) 54(11):4278–87. 10.1007/s10803-023-06140-1 37751100

[21] Bekes K Solanke C Waldhart T Priller J Stamm T. Effect of method of administration on the oral health-related quality of life assessment using the early childhood oral health impact scale (ecohis-G). Clin Oral Investig. (2021) 25(8):5061–6. 10.1007/s00784-021-03818-7 33575885 PMC8342363

[22] John MT Omara M Su N List T Sekulic S Häggman-Henrikson B Recommendations for use and scoring of oral health impact profile versions. J Evid Based Dent Pract. (2022) 22(1):101619. 10.1016/j.jebdp.2021.101619 35219460 PMC8886153

[23] Ghanghas M Manjunath BC Kumar A Shyam R Phogat R Panghal V. Validation of the hindi version of the early childhood oral health impact scale among 3-5-year-old preschool children in rohtak city, haryana. J Indian Soc Pedod Prev Dent. (2019) 37(4):333–8. 10.4103/JISPPD.JISPPD_128_18 31710006

[24] Martins-Junior PA Ramos-Jorge J Paiva SM Marques LS Ramos-Jorge ML. Validations of the Brazilian version of the early childhood oral health impact scale (ecohis). Cad Saude Publica. (2012) 28(2):367–74. 10.1590/s0102-311x2012000200015 22331162

[25] Abanto J Paiva SM Sheiham A Tsakos G Mendes FM Cordeschi T Changes in preschool children’s Ohrqol after treatment of dental caries: responsiveness of the B-ecohis. Int J Paediatr Dent. (2016) 26(4):259–65. 10.1111/ipd.12192 26370072

[26] Bekes K Omara M Safar S Stamm T. The German version of early childhood oral health impact scale (ecohis-G): translation, reliability, and validity. Clin Oral Investig. (2019) 23(12):4449–54. 10.1007/s00784-019-02893-1 30993536

[27] Peker K Uysal O Bermek G. Cross—cultural adaptation and preliminary validation of the turkish version of the early childhood oral health impact scale among 5-6-year-old children. Health Qual Life Outcomes. (2011) 9:118. 10.1186/1477-7525-9-118 22192577 PMC3310831

[28] Lee GH McGrath C Yiu CK King NM. Translation and validation of a Chinese language version of the early childhood oral health impact scale (ecohis). Int J Paediatr Dent. (2009) 19(6):399–405. 10.1111/j.1365-263X.2009.01000.x 19811551

[29] Hashim AN Yusof ZY Esa R. The malay version of the early childhood oral health impact scale (malay-ecohis)-assessing validity and reliability. Health Qual Life Outcomes. (2015) 13:190. 10.1186/s12955-015-0386-2 26607665 PMC4660630

[30] Zaror C Atala-Acevedo C Espinoza-Espinoza G Munoz-Millan P Munoz S Martinez-Zapata MJ Cross-cultural adaptation and psychometric evaluation of the early childhood oral health impact scale (ecohis) in Chilean population. Health Qual Life Outcomes. (2018) 16(1):232. 10.1186/s12955-018-1057-x 30554568 PMC6296046

[31] del Carmen Aguilar-Diaz F Irigoyen-Camacho ME. Validation of the Cpq8-10esp in Mexican school children in urban areas. Med Oral Patol Oral Cir Bucal. (2011) 16(3):e430–5. 10.4317/medoral.16.e430 20711140

[32] Pentapati KC Chenna D Kumar VS Kumar N Pai M Kumar S. Early childhood oral health impact scale (ecohis) questionnaire: reliability generalization meta-analysis of cronbach’s alpha. BMC Oral Health. (2025) 25(1):947. 10.1186/s12903-025-06342-2 40490759 PMC12150495

[33] Knorst JK Sfreddo CS de FMG Zanatta FB Vettore MV Ardenghi TM. Socioeconomic status and oral health-related quality of life: a systematic review and meta-analysis. Community Dent Oral Epidemiol. (2021) 49(2):95–102. 10.1111/cdoe.12616 33368600

[34] Almajed OS Aljouie AA Alharbi MS Alsulaimi LM. The impact of socioeconomic factors on pediatric oral health: a review. Cureus. (2024) 16(2):e53567. 10.7759/cureus.53567 38445162 PMC10914081

[35] Aimee NR Dame-Teixeira N Alves LS Borges GA Foster Page L Mestrinho HD Responsiveness of oral health-related quality of life questionnaires to dental caries interventions: systematic review and meta-analysis. Caries Res. (2019) 53(6):585–98. 10.1159/000500855 31280258

[36] Frencken JE Peters MC Manton DJ Leal SC Gordan VV Eden E. Minimal intervention dentistry for managing dental caries—a review: report of a fdi task group. Int Dent J. (2012) 62(5):223–43. 10.1111/idj.12007 23106836 PMC3490231

[37] Schwendicke F Frencken JE Bjorndal L Maltz M Manton DJ Ricketts D Managing carious lesions: consensus recommendations on carious tissue removal. Adv Dent Res. (2016) 28(2):58–67. 10.1177/0022034516639271 27099358

[38] Schwendicke F Splieth C Breschi L Banerjee A Fontana M Paris S When to intervene in the caries process? An expert delphi consensus statement. Clin Oral Investig. (2019) 23(10):3691–703. 10.1007/s00784-019-03058-w 31444695

[39] Oliveira MA Vale MP Bendo CB Paiva SM Serra-Negra JM. Influence of negative dental experiences in childhood on the development of dental fear in adulthood: a case-control study. J Oral Rehabil. (2017) 44(6):434–41. 10.1111/joor.12513 28386938

[40] Godois LDS Knorst JK Noronha TG Emmanuelli B Ardenghi TM Tomazoni F. Pathways to dental fear from childhood to adolescence: a 10-year cohort study. Int J Paediatr Dent. (2023) 33(6):553–62. 10.1111/ipd.13065 36939652

[41] Duangthip D Chen KJ Gao SS Lo ECM Chu CH. Managing early childhood caries with atraumatic restorative treatment and topical silver and fluoride agents. Int J Environ Res Public Health. (2017) 14(10):1204. 10.3390/ijerph14101204 28994739 PMC5664705

[42] Pascareli-Carlos AM Martins LF Silva Gonçalves MdS P Imparato JC Tedesco TK. Pain perception of children after restorative treatments: atraumatic restorative treatment versus chemomechanical removal—a noninferiority randomized clinical trial. J Indian Soc Pedod Prev Dent. (2021) 39(2):202–7. 10.4103/jisppd.jisppd_426_20 34341242

[43] Novaes TF Pontes LRA Freitas JG Acosta CP Andrade KCE Guedes RS Responsiveness of the early childhood oral health impact scale (ecohis) is related to dental treatment complexity. Health Qual Life Outcomes. (2017) 15(1):182. 10.1186/s12955-017-0756-z 28931398 PMC5608161

[44] Alsumait A ElSalhy M Amin M. Long-term effects of school-based oral health program on oral health knowledge and practices and oral health-related quality of life. Med Princ Pract. (2015) 24(4):362–8. 10.1159/000430096 26045154 PMC5588237

[45] Ruff RR Barry-Godin T Niederman R. Effect of silver diamine fluoride on caries arrest and prevention: the cariedaway school-based randomized clinical trial. JAMA Netw Open. (2023) 6(2):e2255458. 10.1001/jamanetworkopen.2022.55458 36757696 PMC9912124

[46] Crystal YO Niederman R. Evidence-based dentistry update on silver diamine fluoride. Dent Clin North Am. (2019) 63(1):45–68. 10.1016/j.cden.2018.08.011 30447792 PMC6500430

[47] Askar H Schwendicke F Lausch J Meyer-Lueckel H Paris S. Modified resin infiltration of non-, micro- and cavitated proximal caries lesions *in vitro*. J Dent. (2018) 74:56–60. 10.1016/j.jdent.2018.03.010 29775637

[48] Dorri M Dunne SM Walsh T Schwendicke F. Micro-invasive interventions for managing proximal dental decay in primary and permanent teeth. Cochrane Database Syst Rev. (2015) 2015(11):CD010431. 10.1002/14651858.CD010431.pub2 26545080 PMC8504982

[49] Arslan S Kaplan MH. The effect of resin infiltration on the progression of proximal caries lesions: a randomized clinical trial. Med Princ Pract. (2020) 29(3):238–43. 10.1159/000503053 31476757 PMC7315193

[50] Jorge RC Ammari MM Soviero VM Souza IPR. Randomized controlled clinical trial of resin infiltration in primary molars: 2 years follow-up. J Dent. (2019) 90:103184. 10.1016/j.jdent.2019.103184 31465818

[51] Paris S Bitter K Krois J Meyer-Lueckel H. Seven-year-efficacy of proximal caries infiltration–randomized clinical trial. J Dent. (2020) 93:103277. 10.1016/j.jdent.2020.103277 31931026

[52] Innes NPT Robertson MD. Recent advances in the management of childhood dental caries. Arch Dis Child. (2018) 103(4):311–5. 10.1136/archdischild-2017-313196 29463521

[53] Carrillo-Diaz M Crego A Armfield JM Romero-Maroto M. Treatment experience, frequency of dental visits, and children’s dental fear: a cognitive approach. Eur J Oral Sci. (2012) 120(1):75–81. 10.1111/j.1600-0722.2011.00921.x 22288924

[54] Barreto KA dos Prazeres LDKT Lima DSM Redivivo RMMP Colares V. Children’s anxiety during dental treatment with minimally invasive approaches: findings of an analytical cross-sectional study. Pesqui Bras Odontopediatria Clin Integr. (2017) 17(1):e3146. 10.4034/PBOCI.2017.171.15

[55] Paris S Meyer-Lueckel H Kielbassa AM. Resin infiltration of natural caries lesions. J Dent Res. (2007) 86(7):662–6. 10.1177/154405910708600715 17586715

[56] Knosel M Eckstein A Helms HJ. Long-term follow-up of camouflage effects following resin infiltration of post orthodontic white-spot lesions *in vivo*. Angle Orthod. (2019) 89(1):33–9. 10.2319/052118-383.1 30324799 PMC8137114

[57] Kim E-Y An U-j Kim S Jeong T-s. Resin infiltration for the esthetic improvement of anterior teeth with developmental defects and post-orthodontic decalcification. J Korean Acad Pediatr Dent. (2010) 37(2):218–24.

[58] dos Santos PGST Silva LB Travassos RMC de Araújo VLC Nunes JHC de Sá Rodrigues VM Minimally invasive dentistry: principles, advances and perspectives for the future. ARACÊ. (2025) 7(6):33681–92. 10.56238/arev7n6-271

[59] Dziaruddin N Zakaria ASI. Resin infiltration of non-cavitated enamel lesions in paediatric dentistry: a narrative review. Children (Basel). (2022) 9(12). 10.3390/children9121893 36553336 PMC9776437

[60] Borges A Caneppele T Masterson D Maia L. Is resin infiltration an effective esthetic treatment for enamel development defects and white spot lesions? A systematic review. J Dent. (2017) 56:11–8. 10.1016/j.jdent.2016.10.010 27793705

[61] Prada AM Potra Cicalău GI Ciavoi G. A review of white spot lesions: development and treatment with resin infiltration. Dent J. (2024) 12(12):375. 10.3390/dj12120375 PMC11674204 39727432

[62] Locker D Slade G. Concepts of oral health, disease and the quality of life. In: Slade GD, editor. Measuring Oral Health and Quality of Life. Chapel Hill (NC): University of North Carolina, Dental Ecology (1997). p. 11–24.

[63] Arrow P Klobas E. Minimal intervention dentistry for early childhood caries and child dental anxiety: a randomized controlled trial. Aust Dent J. (2017) 62(2):200–7. 10.1111/adj.12492 27878824

[64] Al-Halabi M Salami A Alnuaimi E Kowash M Hussein I. Assessment of paediatric dental guidelines and caries management alternatives in the post COVID-19 period. a critical review and clinical recommendations. Eur Arch Paediatr Dent. (2020) 21(5):543–56. 10.1007/s40368-020-00547-5 32557183 PMC7298449

[65] Kramer PF Feldens CA Ferreira SH Bervian J Rodrigues PH Peres MA. Exploring the impact of oral diseases and disorders on quality of life of preschool children. Community Dent Oral Epidemiol. (2013) 41(4):327–35. 10.1111/cdoe.12035 23330729

[66] Barbosa TS Gaviao MB. Oral health-related quality of life in children: part II. Effects of clinical oral health status. a systematic review. Int J Dent Hyg. (2008) 6(2):100–7. 10.1111/j.1601-5037.2008.00293.x 18412721

[67] Perazzo MF Martins-Junior PA Abreu LG Mattos FF Pordeus IA Paiva SM. Oral health-related quality of life of pre-school children: review and perspectives for new instruments. Braz Dent J. (2020) 31(6):568–81. 10.1590/0103-6440202003871 33237227

[68] Srivastava M Schroth RJ Sareen S Lee VHK Cruz de Jesus V Mittermuller BA Oral health-related quality of life of children following different treatment regimens of silver diammine fluoride. J Dent Child (Chic). (2024) 91(3):137–45. PMID: 39743577. 39743577

[69] Quritum M Abdella A Amer H El Tantawi M. Effect of silver diamine fluoride and nano silver fluoride on oral health-related quality of life of children with early childhood caries: a randomized clinical trial. J Dent. (2024) 142:104878. 10.1016/j.jdent.2024.104878 38311016

[70] Gallo MJD Almeida Dos Santos TC Souza Ramos AB De Rossi A de Paula-Silva FWG Borelli Neto L Impact of resin infiltration on self-reported bullying and ohrqol in children with mih: a single-arm clinical trial. Eur Arch Paediatr Dent. (2026):1–10. 10.1007/s40368-026-01176-0 41766023

[71] Watt RG. Oral health inequalities—developments in research, policy and practice over the last 50 years. Community Dent Oral Epidemiol. (2023) 51(4):595–9. 10.1111/cdoe.12880 37243417

[72] Peres MA Macpherson LMD Weyant RJ Daly B Venturelli R Mathur MR Oral diseases: a global public health challenge. Lancet. (2019) 394(10194):249–60. 10.1016/S0140-6736(19)31146-8 31327369

[73] Martignon S Guarnizo-Herreno CC Franco-Cortes AM Garcia-Zapata LM Ochoa-Acosta EM Restrepo-Perez LF Socioeconomic inequalities in early childhood caries: evidence from vulnerable populations in Colombia. Braz Oral Res. (2024) 38:e126. 10.1590/1807-3107bor-2024.vol38.0126 39661799 PMC11654886

[74] Schwendicke F Bombeck L. Cost-effectiveness of school-based caries screening using transillumination. J Dent. (2023) 137:104635. 10.1016/j.jdent.2023.104635 37541420

[75] Davidson T. Perspective chapter: cost-effectiveness of caries preventive programs. In: Chibinski ACR, editor. Dental Caries Perspectives—a Collection of Thoughtful Essays. London: IntechOpen (2023).

[76] Ashley PF Williams C Moles DR Parry J. Sedation versus general anaesthesia for provision of dental treatment to patients younger than 18 years. Cochrane Database Syst Rev. (2015) (9):CD006334. 10.1002/14651858.CD006334.pub4 26413895 PMC7387131

[77] Webbe J Sinha I Gale C. Core outcome sets. Arch Dis Child Educ Pract Ed. (2018) 103(3):163–6. 10.1136/archdischild-2016-312117 28667046

[78] Kortbeek S Pawaria A Ng VL. Equivalence of paper and electronic-based patient reported outcome measures for children: a systematic review. J Pediatr Gastroenterol Nutr. (2023) 76(2):128–36. 10.1097/MPG.0000000000003636 36240491

[79] Schwendicke F Samek W Krois J. Artificial intelligence in dentistry: chances and challenges. J Dent Res. (2020) 99(7):769–74. 10.1177/0022034520915714 32315260 PMC7309354

